# 659 Assessing Emergency Medical Service Handoff and Resuscitation Metrics for Burn Injuries Using Video Review

**DOI:** 10.1093/jbcr/iraf019.288

**Published:** 2025-04-01

**Authors:** Karen Irizarry, Victoria Miles, Bahaa Succar, Erin Adams, Ashley Sanders, Ryan Dumas, Samuel Mandell

**Affiliations:** Parkland Burn Center; University Medical Center New Orleans Burn Center; University of Texas Southwestern; University of Texas Southwestern; Parkland Health; University of Texas Southwestern Medical Center; University of Texas Southwestern / Parkland

## Abstract

**Introduction:**

Utilization of trauma video review for quality and performance improvement initiatives has increased in recent years across numerous institutions. The value of video review for burn resuscitations remains unknown. The Joint Commission estimates over 80% of serious, preventable adverse medical events occur due to ineffective handoffs. We sought to study emergency medical service (EMS) handoff and measure initial resuscitation metrics using burn resuscitation video review (BVR). This initial review sought to determine the feasibility of using BVR to study the compliance of EMS providers and outside hospitals with Advanced Burn Life Support (ABLS) guidelines.

**Methods:**

As part of an ongoing multi-center study of BVR at ABA verified burn centers, we reviewed 15 burn resuscitations from a single institution between May 2024 – August 2024. A team of five analysts reviewed the recorded resuscitations. EMS handoffs were evaluated in terms of content, interruptions, and overall efficacy to include the age, sex, weight, and past medical history of the patient, time of injury, possibility of inhalation injury, most recent vital signs, medical treatments prior to arrival, estimated total body surface area of the burn, hourly urine output, and hourly fluid titration. Data collection includes time to initiation of resuscitation and initial burn treatment.

**Results:**

Video review of burn resuscitations and EMS handoff is feasible, and the audiovisual clarity is sufficient for data extraction. EMS handoff, measured from the time the EMS provider started speaking until report completion, including answered questions, had an average length of [0:01:28 minutes] with a range of [0:00:27 – 0:03:30 minutes]. Interruptions during handoff were seen in all encounters (15/15). Content of handoff varied with 100% including mechanism of injury, 60% including age, and 33.3% including time of injury. When looking at vital signs, most recent heart rate was communicated in 26% of handoffs and blood pressure in 40%, whereas temperature was not relayed in any case. Inconsistencies in initial burn resuscitations were also seen, with 46.6% of patients not receiving any intravenous fluids from EMS and 33.3% of receiving burn teams not specifying fluid resuscitation upon patient arrival.

**Conclusions:**

Preliminary analysis of this ongoing project indicates BVR will allow for effective quality improvement efforts targeting EMS handoff and resuscitation by evaluating real-time adherence rates to current ABLS guidelines and the initial resuscitation of our burn patients.

**Applicability of Research to Practice:**

BVR may identify key opportunities for improvement in EMS communication and ABLS guideline adherence. For the first time, we will be able to directly evaluate real-time practice rather than infer from documentation.

**Funding for the Study:**

N/A

